# Association of Protein, Zinc, and Vitamin A Maternal Intake With Preterm Birth, but Not With the Dietary Inflammatory Index

**DOI:** 10.1155/jnme/5387274

**Published:** 2025-12-01

**Authors:** Rima Irwinda, Lisa Novianti, Nadira Afida Kalisya

**Affiliations:** Department of Obstetrics and Gynecology, Faculty of Medicine, Dr. Cipto Mangunkusumo Hospital, University of Indonesia, Jakarta, Indonesia

**Keywords:** dietary inflammatory index, nutrient intake, pregnancy, preterm birth

## Abstract

**Introduction:**

Preterm birth is defined as any birth that occurs before 37 weeks of gestational age. National Basic Health Research 2018 shows that 29.5% of births are preterm in Indonesia. Preterm delivery is associated with various risk factors and etiologies, such as malnutrition, inflammations, infections, pathological uterine distention, stress, and environmental toxins. Maternal diet plays a significant role in regulating chronic inflammation. This study aims to investigate the relationship between the dietary inflammatory index (DII) and preterm birth, as well as measure the nutritional intake of pregnant women.

**Methods:**

This study included 365 pregnant women who showed signs of parturition at Cipto Mangunkusumo General Hospital between June 2021 and July 2022. They were divided into two groups: preterm and term birth. Dietary information was collected using a food frequency questionnaire. Nutrisurvey v2007 was used to convert the data into precise nutritional intake using an Indonesian food database. The DII was calculated based on 26 food parameters, and the participants were divided into tertiles by their DII. The lowest tertile represented the most anti-inflammatory DII, while the highest represented the most proinflammatory DII.

**Results:**

Preterm births were significantly associated with socioeconomic status (odds ratio [OR] = 0.43, 95% confidence interval [CI] = 0.29–0.66, *p*=0.007), fewer antenatal visits (OR = 3.10, 95% CI = 1.79–5.37, *p* < 0.001), inadequate intake of micronutrient supplements (OR = 0.44, 95% CI = 0.19–0.96, *p*=0.035), vaginal bleeding (OR = 2.56, 95% CI = 1.13–5.79, *p*=0.020), maternal energy intake (*p* < 0.001), vitamin B_12_ (*p*=0.031), and amino acids (*p*=0.036). Multivariate analysis showed that fewer antenatal visits (adjusted OR [aOR] = 2.23, 95% CI = 1.34–3.78, *p*=0.001), vaginal bleeding (aOR = 3.76, 95% CI = 1.51–9.33, *p*=0.004), contributed to preterm birth. Lower energy (*p*=0.009) and protein (*p*=0.015) intake were significantly associated with birth outcomes. Higher zinc (*p*=0.041) and vitamin A (*p*=0.006) intake significantly reduced the risk of preterm birth. The DII was not significantly correlated with preterm birth.

**Conclusion:**

Lower antenatal visits, vaginal bleeding, higher energy, higher protein, lower zinc, and lower vitamin A intake were significantly associated with preterm birth. The DII was not significantly correlated with preterm birth.

## 1. Introduction

The World Health Organization (WHO) defines any birth that occurs before 37 weeks of gestational age as preterm. In 2020, 13.4 million infants were born prematurely. Prematurity is a contributing factor in the death of about 900,000 newborns annually, establishing prematurity as the leading cause of death in children aged under five years [1]. National Basic Health Research 2018 showed that 29.5% of births in Indonesia were preterm [2]. Preterm birth is associated with various risk factors and etiologies, such as maternal characteristics, history of preterm birth, and the characteristics of the current pregnancy. Maternal malnutrition, infection, inflammation, hypertension, preeclampsia, premature membrane rupture, and low socioeconomic status are among the risk factors for preterm birth. Most (40%–45%) preterm births are idiopathic and occur spontaneously, while 30% are related to preterm premature rupture of membranes (PPROM), and 15%–20% are related to other indications, such as maternal disease or fetal abnormalities [3–5].

Many theories and hypotheses have attempted to explain the mechanism of spontaneous preterm births, including (1) inflammation of the intra-amniotic, which causes cellular stress and death, initiating uterine contractions, cervical ripening, and membrane rupture; (2) fetal membrane dysfunction, including chorioamnionitis and uterine overdistention, which could disrupt membrane remodeling; and (3) fetal or maternal stress could activate the fetal hypothalamic–pituitary–adrenal axis [6, 7]. Nutrition contributes to pathomechanisms that potentially increase the risk of preterm birth, such as infection, inflammation, oxidative stress, and muscle contractility [8].

Malnutrition, particularly during pregnancy, remains a significant public health concern, especially among women in low- and middle-income countries. This condition is linked to various adverse pregnancy outcomes, including congenital malformations, preterm birth, low birth weight, preeclampsia, gestational diabetes, and intrauterine growth retardation, which is a leading cause of neonatal morbidity and mortality worldwide. Research has shown that insufficient maternal nutrient intake, encompassing both macronutrients and micronutrients, plays a critical role in determining pregnancy outcomes. The failure to maintain a balance between pro-oxidants and antioxidants can result in oxidative stress, leading to premature aging of the placenta and increased inflammation, which is hypothesized to trigger preterm birth [9–11].

Several studies have demonstrated that maternal nutrient intake plays a crucial role in pregnancy outcomes, including preterm birth. A healthy diet, rich in vegetables, fruits, whole grains, fish, and dairy, has been linked to a reduced risk of preterm birth, while high consumption of fast food and saturated fats increases this risk. Folate supplementation before and during pregnancy has also been shown to reduce the risk of preterm birth. Additionally, multivitamin and mineral supplements (MMS) have been associated with improved birth weight and reduced preterm birth rates. Vitamin D deficiency during pregnancy is linked to a higher risk of preeclampsia, gestational diabetes, and preterm birth. These findings emphasize the importance of adequate maternal nutrition in preventing preterm birth and promoting healthy pregnancy outcomes [12].

Malnutrition during pregnancy is still a major problem in Indonesia. National Basic Health Research 2013 showed that 32.9% of women in Indonesia suffer from obesity, and 24.2% of pregnant women experience chronic low energy. Obesity is a known risk factor for preterm birth. Cnattingius et al. [13] described how women with obesity grades 2 and 3 had 0.2%–0.4% higher rates of preterm births. It has also been shown that 48.9% of pregnant women in Indonesia have anemia, with most aged 15–24 years. In recent years, some experts have suggested that the diet of pregnant women can impact both fetal development and pregnancy outcomes, as well as their inflammatory response. The dietary inflammatory index (DII) is an emerging tool used to assess the inflammatory potential of an individual's diet. Unlike conventional measures of nutritional intake, such as total energy or specific nutrient consumption, the DII offers a broader view of dietary patterns by evaluating the overall inflammatory load of the diet [14].

Before the DII was developed, nutritional indices were too specific and not sufficiently broad to encompass the diversity of human populations. This tool was created based on a comprehensive review of studies assessing each food parameter's impact on six inflammatory biomarkers: interleukin (IL)-1β, IL-4, IL-6, IL-10, tumor necrosis factor (TNF)-*α*, and C-reactive protein (CRP) [15]. CRP levels were lower in mothers who ate more fruits, vegetables, fish, and protein than in those who ate a “Western” diet high in red meat, fats, and simple carbohydrates, resulting in higher levels of proinflammatory mediators. A lower DII indicates an anti-inflammatory diet, while a higher DII indicates a proinflammatory diet. The DII has been validated in various populations, including both pregnant and non-pregnant individuals worldwide. This makes it a unique and potentially more effective predictor of inflammatory-related pregnancy outcomes, such as preterm birth or preeclampsia [16, 17].

Despite its growing use in nutrition research, the association of the DII with pregnancy outcomes, particularly preterm birth, in pregnant women in developing countries has not been fully explored. This study aims to fill this gap by examining the relationship between maternal nutrient intake, the DII, and preterm birth. By focusing on both macronutrient and micronutrient sufficiency, as well as the inflammatory potential of the diet, this research intends to contribute to a deeper understanding of how dietary patterns influence preterm birth and to evaluate whether the DII could serve as a more precise tool than traditional nutritional metrics.

## 2. Methods

### 2.1. Study Design and Participants

**Figure 1:**
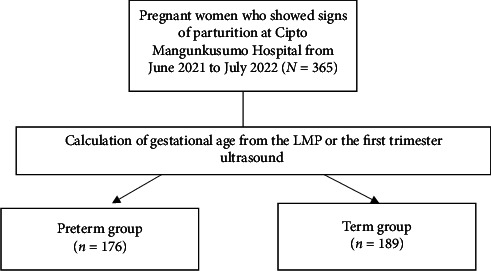
Participant flowchart.

This case–control study was conducted between June 2021 and July 2022 at Cipto Mangunkusumo General Hospital, a tertiary national referral hospital. It included 365 pregnant women who showed signs of parturition (first or second stage of labor) and were divided into two groups: preterm and term. The preterm group (*n* = 176) consisted of pregnant women who delivered live births at 22^+0^–36^+6^ weeks of gestation, and the control group (*n* = 189) consisted of pregnant women who delivered live births ≥ 37^+0^ weeks. Pregnant women with congenital fetal anomalies were excluded. The gestational age was calculated from the last menstrual period (LMP) during history taking or the precise date recorded in the first-trimester ultrasound recorded in the antenatal book. Following the history taking and physical examination, an additional ultrasound was conducted to verify the gestational age and to finalize the data in advance. This study was approved by the Ethical Committee for Research in Human of the Faculty of Medicine, Universitas Indonesia (KET-553/UN2.F1/ETIK/PPM.00.02/2021). All 365 participants provided informed consent for this study. The participant flowchart is shown in Figure 1.

### 2.2. Data Collection and Measurements

We collected clinical characteristics through history taking, physical examinations, laboratory tests, and review of medical records. Maternal characteristics such as maternal age, BMI, comorbidities (hypertension and diabetes mellitus), smoking, history of surgery, parity, socioeconomic status, interpregnancy intervals, multifetal pregnancy, the number of antenatal visits, maternal infection, anemia, history of hyperemesis gravidarum, and vaginal bleeding were collected in the case report form (CRF).

The cutoff for anemia in this study follows the WHO classification, defined as hemoglobin levels < 11 g/dL in the first and third trimesters and < 10.5 g/dL in the second trimester. Vaginal bleeding is defined as bleeding from the birth canal during the first or second trimester of pregnancy.

Abnormal amniotic fluid was assessed via using ultrasound and/or data from the latest maternal–fetal ultrasound documented in medical records.

### 2.3. Assessment of Dietary Intake

All participants completed a one-month semiquantitative food frequency questionnaire (FFQ). The FFQ was administered once during pregnancy at the time of the participant's hospital visit. Since the timing of administration depended on when each participant enrolled in the study, the FFQ captured dietary intake from varying gestational periods among participants.

The FFQ was conducted by a trained and certified nutritionist using food models. Then, the data were converted into precise nutritional intake using Nutrisurvey (Version 2007) with an Indonesian food database. Nutritional values are categorized as low, normal, and high based on international recommendations by the Institute of Medicine (IOM) dietary reference intakes (DRIs). For nutrients without IOM references, we referred to previously published peer-reviewed studies.

### 2.4. DII Calculation

The DII was calculated using the method of Shivappa et al. [18]. Data were calculated for each parameter based on the dietary intake using a worldwide database. This assessment is based on 1943 articles that examined the relationship between 45 dietary parameters and one of six inflammatory factors: IL-1β, IL-6, TNF-α, or CRP. The values are assigned +1 if there is a significant increase, −1 if there is a significant decrease, and 0 if there is no change in inflammatory factors. The number of food parameters used in this study was adjusted according to the data obtained in the field.

This study used 26 of the 45 food parameters derived from the FFQ to calculate the DII: energy, carbohydrates, protein, total fat, omega 3, omega 6, polyunsaturated fatty acids (PUFAs), monounsaturated fatty acids (MUFAs), cholesterol, trans fat, fiber, β-carotene, thiamine, riboflavin, niacin, isoflavone, folic acid, vitamin A, vitamin B_6_, vitamin B_12_, vitamin C, vitamin D, vitamin E, iron, magnesium, and zinc. Some food parameters that are rarely used in Indonesia, such as saffron, anthocyanidin, and rosemary, were excluded from analysis.

Total energy expenditure (TEE) was used to determine the individualized energy requirement range for each participant. TEE was estimated based on the Institute of Medicine (IOM) equation for estimated energy requirement (EER). TEE (kcal/day) formula for pregnant women aged 19 years or older = 354 − (6.91 × age [years]) + PA × [(9.36 × weight [kg]) + (726 × height [m])] + additional calories according to gestational age (+340 kcal/day in the second trimester, +452 kcal/day in the third trimester). Physical activity (PA) levels were not individually assessed; therefore, a standard PA coefficient of 1.0 (sedentary) was applied to all participants. Energy values were expressed in kilocalories per day (kcal/day).

A *Z*-score was calculated by subtracting the nutrient intake amount from the global mean and dividing the result by the global standard deviation (SD). After calculating the *Z*-score for each nutrient intake, we converted it into a percentile score to minimize its right skew. Each percentile score was converted to a centered percentile score to achieve a symmetrical distribution. The final DII amount of each parameter was calculated by multiplying the centered percentile score by the “overall inflammatory effect score” introduced by Shivappa et al. [18].

### 2.5. Statistical Analysis

The statistical analyses were performed using IBM SPSS Statistics (Version 27). Normally distributed continuous variables are summarized as the mean and SD, while non-normally distributed variables are summarized as the median (range). Numerical variables were compared between groups using a Mann–Whitney *U* test, and categorical variables were compared using a chi-square test. Variables with a *p* < 0.25 in bivariate analysis were included in the multivariate logistic regression model. Odds ratios (ORs) with 95% confidence intervals (CIs) were calculated using the final multivariate model. Statistical significance was set at *p* < 0.05 for the bivariate and multivariate analysis.

The use of a *p* value cutoff of < 0.25 to identify variables as potential candidates for bivariate or multivariate analysis is grounded in the study by Hosmter et al. [19] This study is also consistent with the study by Bursac et al. [20] that indicated that employing a traditional *p* value cutoff (*p* < 0.05) often excludes variables that may ultimately prove significant. Therefore, utilizing a higher *p* value cutoff can help ensure that crucial and potential predictors are included in the model.

Some variables were converted into dichotomous variables: maternal age (< 35 and ≥ 35 years old), socioeconomic status (low and middle income and high and very high income), interpregnancy intervals, and the number of antenatal visits (< 7 and ≥ 7 antenatal visits).

## 3. Results

This study examined the data of 365 participants, of which 189 had term births and 176 had preterm births. Participants' characteristics are presented in Table 1. Preterm births were significantly associated with socioeconomic status (OR = 0.43, 95% CI = 0.29–0.66, *p*=0.007), fewer antenatal visits (OR = 3.10, 95% CI = 1.79–5.37, *p* < 0.001), inadequate intake of micronutrient supplements (OR = 0.44, 95% CI = 0.19–0.96, *p*=0.035), and vaginal bleeding (OR = 2.56, 95% CI = 1.13–5.79, *p*=0.020).

The daily nutrition intake and nutrient status of the 365 participants were assessed (Table 2). Almost all participants (93.4%) had a low iron intake, and most also had low intakes of protein (56.4%), zinc (77.8%), magnesium (65.8%), folic acid (97.8%), vitamin C (65.8%), vitamin D (97.8%), vitamin E (94.8%), vitamin B_6_ (82.2%), PUFAs (53.4%), MUFAs (98.9%), omega-6 (92.1%), fiber (91.0%), β-carotene (100%), thiamine (86.0%), riboflavin (60.3%), niacin (97.8%), vitamin K (88.5%), copper (100%), and sodium (100%). Based on the recommended daily allowances for Indonesians, 44.9% of the participants had a low daily energy intake, with a median of 2361.6 kcal/day. Based on the DRIs, most participants had higher intakes of carbohydrates (57.0%), total fat (100%), vitamin A (62.5%), and cholesterol (74.5%). However, most participants had sufficient intakes of vitamin B_12_ (75.6%) and amino acids (78.4%).

The associations between participants' daily nutrition intake and pregnancy outcomes are shown in Table 3. The parameters significantly associated with pregnancy outcomes were energy (*p* < 0.001), vitamin B_12_ (*p*=0.031), and amino acids (*p*=0.036).

Table 4 shows the participants' DII divided into four quartiles. Quartile 1 (Q1) represents the most anti-inflammatory DII, and quartile 4 (Q4) represents the most proinflammatory DII. Among the participants, 27.9% had a DII in Q4, and 25.8% had a DII in Q1. The median DII was −0.133. The DII was not significantly correlated with preterm birth (Table 5).

Table 6 presents the ORs from bivariate logistic regressions of variables that differed significantly between the term and preterm groups.

Maternal characteristics, including the number of antenatal visits (adjusted OR [aOR] = 2.23, 95% CI = 1.34–3.78, *p*=0.001) and vaginal bleeding (aOR = 3.76, 95% CI = 1.51–9.33, *p*=0.004) were identified as significantly associated with preterm birth (Table 6). In addition, maternal nutrient intake, including lower energy (aOR = 1.02, 95% CI = 1.01–1.03, *p*=0.009) and protein aOR = 2.11, 95% CI = 1.15–3.85, *p*=0.015) intake was significantly associated with preterm birth. In addition, higher zinc (aOR = 0.38, 95% CI = 0.15–0.96, *p*=0.041) and vitamin A (aOR = 0.48, 95% CI = 0.26–0.82, *p*=0.006) intake reduced the risk of preterm birth.

## 4. Discussion

Our study found several significant factors associated with the risk of preterm birth, including antenatal care (ANC) visits, vaginal bleeding, and maternal nutrient intake such as energy, protein, zinc, and vitamin A. These findings highlight the complex interplay of clinical and dietary factors that contribute to preterm birth in our study population.

The number of antenatal visits was correlated with preterm birth in our study (*p*=0.001). Pervin et al. [21] reported a similar finding, showing that a lower number of ANC visits was linked to a higher risk of preterm birth. Specifically, preterm birth occurred 2.4 times more frequently among women who attended ≤ 1 ANC visit compared to those who had ≥ 3 visits (OR = 2.37, 95% CI = 2.07–2.70). The importance of ANC in reducing the likelihood of preterm birth is early identification and management of risk factors, such as previous preterm births, multiple pregnancies, anemia, diabetes, hypertensive disorders, and infections, therefore contributing to a reduction in preterm birth rates.

Our findings aligns with a cohort study by Liu [22], which examined 10,179 pregnant women. Among them, 1001 experienced vaginal bleeding during the first trimester, and 119 had preterm births. The study concluded that vaginal bleeding in the first trimester is an independent risk factor for preterm birth, increasing the overall risk by 29% (OR = 1.2, 95% CI = 1.04–1.60). This may be associated with the production of thrombin during vaginal bleeding that cause uterine contraction. Severe or prolonged vaginal bleeding can also result in sub-chorionic hematoma, which increases the risk of placenta previa, pregnancy induced-hypertension, and placental abruption. These conditions are major contributors to preterm birth.

Our study found that lower energy and protein intake was significantly associated with preterm birth. This aligns with findings from other studies, such as the case–control study by Awasthi et al. [23], which reported that energy and protein maternal intake were lower in women who delivered preterm neonates. Lower maternal protein intake is consistently associated with higher odds of preterm birth, plausibly through constrained placental growth, compromised fetal membrane integrity, and dysregulated inflammatory and endocrine signaling. Across observational studies, women who deliver preterm report lower protein consumption, and the association often persists after adjusting for total energy and key confounders, suggesting a protein-specific effect rather than generalized undernutrition. Although our study differs in setting and methods (hospital-based Indonesian case–control with a specific confounder set), it still found that lower maternal protein intake was independently linked to preterm birth after adjustment (*p*=0.015), indicating that protein itself contributes to preterm risk.

Figa et al. [24] conducted a cohort study that revealed undernourished women had a 1.8 times higher risk of preterm birth compared to women with adequate nutrition. Maternal chronic malnutrition could lead to stress, thus leading to maternal adrenal gland production increases of cortisol and oxytocin. These productions increase prostaglandin and G-protein coupled receptors, signaling the influx of intracellular calcium. The huge influx of intracellular calcium could lead to preterm contraction of the uterus and hereby lead to preterm birth [25].

In addition, our study showed that higher zinc and vitamin A intake were associated with reduced risk of preterm birth. Zinc plays a key role in the immune system. Deficiency may result in a weakened immune response, increasing the likelihood of infections, which could cause preterm birth. Zinc deficiency could also disrupt placental development and affect fetal growth, leading to potential birth outcomes, such as preterm birth. A systematic review by Chaffee BW et al. [26] involving 8897 pregnant women reported that preterm birth was associated with lower maternal zinc serum levels. The aOR for lower zinc intake quartiles compared to the highest quartile were 1.29, 1.55, and 1.20, respectively, indicating significantly increased risks of preterm birth. The study by Irwinda et al. [27] highlights the critical role of zinc in late pregnancy, especially in preventing preterm birth and fetal growth restriction (FGR). According to the study, maternal serum zinc levels were significantly lower in cases of preterm birth, suggesting that zinc deficiency may play a role in triggering preterm labor. This underscores the importance of maintaining adequate zinc levels during pregnancy to reduce the risk of adverse outcomes such as preterm birth and FGR.

Higher maternal intake in this study was associated with reduced risk of preterm birth. Study from Vitamin A is crucial for pregnancy as it supplies the fetal reserve and supports maternal metabolism. The WHO recommends regular daily intake of vitamin A via diet or supplements throughout pregnancy only in regions with widespread vitamin A deficiency. Mezzano et al. [28] showed that prenatal vitamin A deficiency was associated with preterm birth. A study by Irwinda et al. [29] showed that AtRA, a vitamin A derivative, has protective effects in preventing preterm birth. Their study showed the concentration of AtRA was higher in term groups compared to the preterm groups across all samples, including maternal serum (*p* < 0.001), placenta (< 0.001), and cord blood (*p* < 0.001).

The DII was not significantly associated with preterm birth in our study. However, notably, some nutrients were lower in participants with preterm births. This finding is consistent with Buxton et al. [30], who examined 1216 pregnant women in Mexico City, Mexico, and found no significant association between the energy-adjusted DII (E-DII) and preterm birth. They showed linear appearances in all three trimesters related to the E-DII and preterm birth. Another study by Sen et al. [31] involving 1808 mother–child pairs in Massachusetts, United States, found no association between the DII and preterm birth. The OR of preterm birth was 1.06 (95% CI = 0.83–1.36) for < 34 weeks of gestation and 1.10 (95% CI = 0.95–1.28) for 34–37 weeks of gestation.

Although our findings align with several studies, they differ from others. A systematic review by Freitas et al. [32] of eight studies assessing the DII as a predictor of pregnancy outcomes stated that the DII was a predictor of several obstetric complications, including preeclampsia, preterm birth, and breastfeeding failure (mean difference = 0.39, 95% CI = 0.02–0.75, *p*=0.04; OR = 1.24, 95% CI = 1.11–1.40, *p*=0.0002). Of the eight studies analyzed, only Ishibashi et al. [33] predicted preterm birth using the DII. However, their study assessed prepregnancy dietary intake and examined both gestational age and birthweight as outcomes within a cohort design, which may account for differences observed compared to our findings.

In summary, our study highlights the complex relationship between clinical factors and maternal dietary intake in predicting preterm birth. The number of antenatal visits, vaginal bleeding, lower energy, and protein intake were all significant contributors to preterm birth risk. In addition, higher maternal zinc and vitamin A intake were associated with reduced risk of preterm birth. While our findings regarding the DII did not show a significant association, they contribute to the growing body of evidence suggesting that both clinical and dietary factors play crucial roles in preterm birth. These findings underscore the importance of early ANC and adequate maternal nutrition in reducing the risk of preterm birth.

### 4.1. Strengths and Limitations

Our study had some strengths and limitations. It presented an in-depth analysis of the nutrient intake in expectant mothers in Jakarta, Indonesia, at both the macronutrient and micronutrient levels. It also developed a standardized DII using a comprehensive FFQ, which is still not commonly used in Indonesia. Therefore, this study may provide a novel tool for calculating the inflammatory potential of food in Indonesia.

While our study provided valuable insights, it also had some limitations that must be acknowledged. Firstly, it could not avoid recall bias, which may have impacted the accuracy of our results. Secondly, its cohort comprised a subset of pregnant women in Jakarta, so its results may not generalize to all pregnant women in Indonesia, especially in rural areas. Thirdly, it did not utilize an objective measure of inflammation, such as a blood test of the CRP level. Fourthly, there were some limitations in determining the inflammatory potential of foods, including no fixed cutoff for the DII, which can vary by food type and other factors consumed. For example, while consuming foods high in fat can increase the DII, simultaneously consuming proinflammatory nutrients may lower the DII.

Further research is needed to determine a simple and effective maternal intake assessment tool for predicting preterm birth.

## 5. Conclusion

The study found that several factors were linked to increased rates of preterm deliveries. A lower number of antenatal visits, vaginal bleeding, and lower maternal energy and protein intake were associated factors linked to the risk of preterm birth. Higher maternal zinc and vitamin A intake were associated with reduced risk of preterm birth. There is no significant correlation between DII and preterm birth in this study. These findings raise questions about the complexity of the factors contributing to preterm birth and suggest that while nutrient intake plays a role, the overall inflammatory response, as measured by the DII, may not be directly linked to the risk of preterm birth in this study population.

## Figures and Tables

**Table 1:** Maternal characteristics.

Variables	Term (*n* = 189)	Preterm (*n* = 176)	OR (95% CI)	*p* value
Clinical characteristics				
Age, median (min–max)	29.0 (16.0–41.0)	30.0 (16.0–45.0)		0.517
Age groups, *n* (%)^†^				
< 18 years old	3 (1.6)	5 (2.8)	1.04 (0.63–1.72)	0.202^∗^
18–35 years old	155 (82.0)	131 (74.4)		
> 35 years old	31 (16.4)	40 (22.8)		
BMI category, *n* (%)^‡^				
Underweight	19 (10.1)	13 (7.4)	0.99 (0.63–1.57)	0.640
Normal	32 (16.9)	37 (21.0)		
Overweight	23 (12.2)	23 (13.1)		
Obese	115 (60.8)	103 (58.5)		
Hypertension, *n* (%)				
Yes	32 (16.9)	43 (24.4)	0.63 (0.40–1.05)	0.076^∗^
No	157 (83.1)	133 (75.6)		
Diabetes mellitus, *n* (%)				
Yes	11 (5.8)	10 (5.7)	1.03 (0.43–2.48)	0.955
No	178 (94.2)	166 (94.3)		
Smoking, *n* (%)				
Yes	0 (0)	0 (0)	—	—
No	189 (100)	176 (100)		
History of surgery, *n* (%)				
Yes	157 (83.1)	151 (85.8)	1.23 (0.79–2.17)	0.473
No	32 (16.9)	25 (14.2)		
Parity				
Nullipara, *n* (%)	73 (38.6)	52 (29.5)	0.66 (0.43–1.03)	0.068^∗^
Multipara, *n* (%)	116 (61.4)	124 (70.5)		
Socioeconomic status, *n* (%)^§^				
Low income	21 (11.1)	34 (19.3)	0.43 (0.29–0.66)	0.007^∗^
Middle income	54 (28.6)	69 (39.2)		
High income	98 (51.9)	60 (34.1)		
Very high income	16 (8.5)	13 (7.4)		
Num. of antenatal visits, *n* (%)^¶^				
≤ 3 visits	5 (2.6)	15 (8.5)	3.10 (1.79–5.37)	< 0.001^∗^
4–6 visits	17 (9.0)	36 (20.5)		
≥ 7 visits	167 (88.4)	125 (71.0)		
Interpregnancy intervals, *n* (%)^††^				
Interval less than 18 months	83 (43.9)	69 (39.2)	1.11 (0.73–1.67)	0.441
18–59 months	60 (31.7)	54 (30.7)		
> 59 months	46 (24.3)	53 (30.1)		
Multifetal pregnancy, *n* (%)				
Yes	4 (2.1)	10 (5.7)	2.78 (0.86–9.05)	0.076^∗^
No	185 (97.9)	166 (94.3)		
Nutritional status				
Balance diet, *n* (%)				
Yes	31 (16.4)	29 (16.5)	0.99 (0.57–1.73)	0.985
No	158 (83.6)	147 (83.5)		
Micronutrient supplementation, *n* (%)				
Yes	179 (94.7)	156 (88.6)	0.44 (0.19–0.96)	0.035^∗^
No	10 (5.3)	20 (11.4)		
Coffee consumption, *n* (%)				
Yes	3 (1.6)	3 (1.7)	1.07 (0.21–5.40)	0.930
No	186 (98.4)	173 (98.3)		
Pregnancy comorbid				
Maternal infection, *n* (%)				
Yes	37 (19.6)	49 (27.8)	1.59 (0.97–2.58)	0.063^∗^
No	152 (80.4)	127 (72.2)		
Anemia, *n* (%)^‡‡^				
Yes	34 (18.0)	25 (14.2)	0.76 (0.43–1.33)	0.326
No	155 (82.0)	151 (85.8)		
Hyperemesis, *n* (%)				
Yes	62 (32.8)	59 (33.5)	1.03 (0.67–1.59)	0.884
No	127 (67.2)	117 (66.5)		
Abnormal amniotic fluid, *n* (%)^§§^				
No	120 (63.5)	108 (61.4)	1.22 (0.77–1.93)	0.713
Oligohydramnios	64 (33.9)	65 (36.9)		
Polyhydramnios	5 (2.6)	3 (1.7)		
Vaginal bleeding, *n* (%)				
Yes	9 (4.8)	20 (11.4)	2.56 (1.13–5.79)	0.020^∗^
No	180 (95.2)	156 (88.6)		

*Note:* Min: minimum; Max: maximum.

Abbreviations: BMI, body mass index; OR, odds ratio.

^∗^ *p* value threshold of < 0.25 was used to identify candidate variables for multivariate logistic regression or a *p* value < 0.05 was used to identify statistical significance.

^†^Age was dichotomized into < 35 vs. ≥ 35 years old.

^‡^BMI was dichotomized into underweight and normal weight vs. overweight and obese.

^§^Socioeconomic status was dichotomized into low- and middle-income vs. high- and very high-income.

^¶^The number of antenatal visits was dichotomized into < 7 visits vs. ≥ 7 visits.

^††^The interpregnancy interval was dichotomized into no interval or < 18 months versus ≥ 18 months.

^‡‡^Anemia is defined as Hb < 11.0 g/dL in the first and third trimesters and < 10.5 g/dL in the second trimester.

^§§^Abnormal amniotic fluid was dichotomized into no abnormality vs. oligohydramnios or polyhydramnios.

**Table 2:** Daily nutrition intake and nutrient status.

Daily nutrient intake	Range	Median (min–max)	*N* (%)
Energy		2361.6 (1806.4–3233.2)	
Low	Calculated based on the total energy expenditure (TEE) of each subject (kcal/day)		164 (44.9)
Normal			201 (55.1)
Carbohydrate		275.6 (1.9–1075.1)	
Low	< 175 g/day		31 (8.5)
Normal	175–265 g/day		126 (34.5)
High	> 265 g/day		208 (57.0)
Protein		69.7 (5.6–378.7)	
Inadequate	< 1.1 g/kg/day		220 (60.3)
Adequate	≥ 1.1 g/kg/day		145 (39.7)
Total fat		118.6 (35.1–380)	
Low	< 25% of calorie intake		0
Normal	25%–35% of calorie intake		0
High	> 35% of calories intake		365 (100)
Fe		13.0 (3–205.9)	
Low	< 27 mg/day		341 (93.4)
Normal	27–45 mg/day		16 (4.4)
High	> 45 mg/day		8 (2.2)
Zinc		8.4 (2.5–192.7)	
Low	< 11 mg/day		284 (77.8)
Normal	11–40 mg/day		77 (21.1)
High	> 40 mg/day		4 (1.1)
Magnesium		297.3 (22.9–1769.9)	
Low	< 350 mg/day		239 (65.5)
Normal	350–400 mg/day		33 (9.0)
High	> 400 mg/day		93
Folic acid		210.3 (4.6–1727.7)	
Low	< 600 μg/day		357 (97.8)
Normal	600–1000 μg/day		6 (1.6)
High	> 1000 μg/day		2 (0.5)
Vitamin A		3,556,100 (87.1–16,023.0)	
Low	< 0.7 mg/day		3 (0.8)
Normal	0.7–3 mg/day		134 (36.7)
High	> 3 mg/day		228 (62.5)
Vitamin C		69.2 (5.3–646.2)	
Low	< 85 mg/day		240 (65.8)
Normal	85–2000 mg/day		125 (34.2)
High	≥ 2000 mg/day		0
Vitamin D		4.5 (0.1–2025.7)	
Low	< 15 μg/day		357 (97.8)
Normal	15–100 μg/day		4 (1.1)
High	> 100 μg/day		4 (1.1)
Vitamin E		8.5 (2.6–78.9)	
Low	< 15 mg/day		345 (94.8)
Normal	15–1000 mg/day		20 (5.2)
High	> 1000 mg/day		0
Vitamin B6		1.4 (0.3–173.3)	
Low	< 1.9 mg/day		300 (82.2)
Normal	1.9–1000 mg/day		65 (17.8)
High	> 1000 mg/day		0
Vitamin B12		4.1 (0.2–117.0)	
Low	< 25 μg/day		359 (98.4)
Normal	≥ 25 μg/day		6 (1.6)
PUFA		16.3 (0.5–261.4)	
Low	< 0.6% of calorie intake		195 (53.4)
Normal	0.6%–1.2% of calorie intake		151 (41.4)
High	> 1.2% of calorie intake		19 (5.2)
MUFA		25.1 (9.6–105.0)	
Low	< 10% of calorie intake		361 (98.9)
Normal	10%–20% of calorie intake		1 (0.3)
High	> 20% of calorie intake		3 (0.8)
Omega-3		300 (0–6000)	
Low	< 250 mg/day		173 (47.4)
Normal	250–300 mg/day		32 (8.8)
High	> 300 mg/day		160 (43.8)
Omega-6		1.3 (0–51.0)	
Low	< 13 g/day		336 (92.1)
Normal	> 13 g/day		29 (7.9)
Cholesterol		274 (32.6–1638.5)	
Normal	< 200 mg/day		93 (25.5)
High	> 200 mg/day		272 (74.5)
Fiber		12.2 (2.4–88.3)	
Low	< 28 g/day		332 (91.0)
Normal	> 28 g/day		33 (9.0)
β-Carotene		0.1 (0–4.8)	
Low	< 0.7 mg/day		302 (82.7)
Normal	0.7–3 mg/day		59 (16.2)
High	> 3 mg/day		4 (1.1)
Thiamine		0.8 (0.2–5.6)	
Low	< 1.4 mg/day		314 (86.0)
Normal	> 1.4 mg/day		51 (14.0)
Riboflavin		1.2 (0.3–9.7)	
Low	< 1.4 mg/day		220 (60.3)
Normal	> 1.4 mg/day		145 (39.7)
Niacin		1.8 (0–40.5)	
Low	< 18 mg/day		357 (97.8)
Normal	18–35 mg/day		7 (1.9)
High	> 35 mg/day		1 (0.3)
Amino acid		102 (58.5–231)	
Inadequate	< 1.5 g/kg/day		365 (100)
Adequate	≥ 1.5 g/kg/day		0
Vitamin K		17.6 (0.1–2025.2)	
Low	< 90 mg/day		323 (88.5)
Normal	> 90 mg/day		42 (11.5)
Copper		1300 (300–8200)	
Low	< 1000 μg/day		365 (100)
Normal	1000–10,000 μg/day		0
High	> 10,000 μg/day		0
Sodium		0.61 (0.05–4.85)	
Low	< 1.5 g/day		359 (98.4)
Normal	1.5–2.3 g/day		4 (1.1)
High	> 2.3 g/day		2 (0.5)

*Note:* Min: minimum; Max: maximum; Fe: ferrum (iron); PUFA: polyunsaturated fatty acid; MUFA: monounsaturated fatty acid; g: gram; mg: milligram; μg: microgram; β-Carotene: beta-carotene. The values are presented as the median (min–max) for numerical variables and *n* (%) for categorical variables.

Abbreviation: TEE, total energy expenditure.

**Table 3:** Association of daily nutrition intake with term and preterm birth.

Daily nutrient intake	Term (*n* = 189)	Preterm (*n* = 176)	OR (95% CI)	*p* value
Energy (kcal/day)	2386.3 (2018.6–3151.3)	2327.8 (1806.4–3233.2)		< 0.001^∗^
Low (*n*, %)	90 (47.6)	74 (42.0)	0.80 (0.53–1.21)	0.285
Normal	99 (52.4)	102 (58.0)		
Carbohydrate (g/day)^†^	278.3 (70.1–1075.1)	271.9 (1.9–586.8)		0.328
Low (*n*, %)	16 (8.5)	15 (8.5)	1.16 (0.77–1.76)	0.486
Normal	62 (32.8)	64 (36.4)		
High	111 (58.7)	97 (55.1)		
Protein (g/day)	71.1 (21.8–378.7)	66.5 (5.6–180.9)		0.795
Inadequate (*n*, %)	121 (64.0)	99 (56.3)	0.72 (0.47–1.1)	0.129^∗^
Adequate	68 (36.0)	77 (43.8)		
Total fat (% of calorie intake)^‡^	120.4 (37.3–380.0)	118.2 (35.1–273.5)		0.965
Low (*n*, %)	0	0	0.95 (1.0–0.99)	—
Normal	0	0		
High	189 (100)	176 (100)		
Fe (mg/day)^§^	12.9 (3.0–191.0)	13.3 (3.8–205.9)		0.612
Low (*n*, %)	178 (94.2)	163 (92.6)	0.78 (0.34–1.78)	0.547
Normal	5 (2.6)	11 (6.3)		
High	6 (3.2)	2 (1.1)		
Zinc (mg/day)^¶^	8.8 (2.5–155.8)	8.3 (3.2–192.7)		0.805
Low (*n*, %)	152 (80.4)	132 (75.0)	0.73 (0.45–1.12)	0.213^∗^
Normal	35 (18.5)	42 (23.9)		
High	2 (1.1)	2 (1.1)		
Magnesium (g/day)^††^	289.9 (82.5–1769.9)	300.6 (22.9–1024.6)		0.562
Low (*n*, %)	121 (64.0)	118 (67.0)	1.14 (0.74–1.76)	0.544
Normal	21 (11.1)	12 (6.8)		
High	47 (24.9)	46 (26.1)		
Folic acid (μg/day)^‡‡^	207.3 (4.6–1362.5)	214.5 (11.7–1727. 7)		0.118^∗^
Low (*n*, %)	185 (97.9)	172 (97.7)	0.93 (0.23–3.78)	0.919
Normal	3 (1.6)	3 (1.7)		
High	1 (0.5)	1 (0.6)		
Vitamin A (μg/day)^§§^	3451.7 (87.1–13,953.0)	3805.4 (144.2–16,023.0)		0.630
Low (n, %)	1 (0.5)	2 (1.1)	0.72 (0.47–1.10)	0.127^∗^
Normal	77 (40.7)	57 (32.4)		
High	111 (58.7)	117 (66.5)		
Vitamin C (mg/day)	69.2 (5.3–646.2)	72.2 (12.5–313.9)		0.680
Low (*n*, %)	130 (68.6)	110 (62.5)	0.72 (0.46–1.14)	0.206^∗^
Normal	59 (31.2)	66 (37.5)		
High	0	0		
Vitamin D (μg/day)^¶¶^	4.6 (0.3–2025.7)	4.5 (0.1–116.7)		0.420
Low (*n*, %)	184 (97.4)	173 (98.3)	1.57 (0.37–6.66)	0.540
Normal	2 (1.1)	2 (1.1)		
High	3 (1.6)	1 (0.6)		
Vitamin E (mg/day)	8.5 (2.6–72.4)	8.4 (3.3–78.9)		0.984
Low (*n*, %)	169 (92.3)	177 (97.3)	1.42 (0.57–3.57)	0.449
Normal	14 (7.7)	5 (2.7)		
High	0	0		
Vitamin B6 (mg/day)	1.4 (0.3–106.1)	1.4 (0.6–173.3)		0.408
Low (*n*, %)	157 (83.1)	143 (81.3)	0.88 (0.52–1.51)	0.650
Normal	32 (16.9)	33 (18.8)		
High	0	0		
Vitamin B12 (μg/day)	3.9 (0.2–72.7)	4.3 (0.4–117)		0.031^∗^
Low (*n*, %)	187 (98.9)	172 (97.7)	0.46 (0.8–2.54)	0.434
Normal	2 (1.1)	4 (2.3)		
PUFA (% of calorie intake)^†††^	16.2 (1.4–99.4)	16.8 (0.5–261.4)		0.564
Low (*n*, %)	95 (50.3)	100 (56.8)	0.77 (0.51–1.17)	0.216^∗^
Normal	84 (44.4)	67 (38.1)		
High	10 (5.3)	9 (5.1)		
MUFA (% of calorie intake)^‡‡‡^	24.8 (9.8–105.0)	25.3 (9.6–61.9)		0.509
Low (*n*, %)	187 (98.9)	174 (98.9)	0.93 (0.13–6.68)	0.943
Normal	0	1 (0.6)		
High	2 (1.1)	1 (0.6)		
Omega-3 (mg/day)^§§§^	200 (0–6000)	300 (0–4500)		0.560
Low (*n*, %)	96 (50.8)	77 (43.8)	0.75 (0.50–1.14)	0.178^∗^
Normal	9 (4.8)	23 (13.1)		
High	84 (44.4)	76 (43.2)		
Omega-6 (g/day)	0.9 (0–51.0)	1.6 (0–38.4)		0.087^∗^
Low (*n*, %)	176 (93.1)	160 (90.9)	0.74 (0.35–1.58)	0.435
Normal	13 (6.9)	16 (9.1)		
Cholesterol (mg/day)	268.1 (32.6–1415.8)	284.1 (81.3–1638.5)		0.490
Normal (*n*, %)	44 (23.3)	49 (27.8)	1.27 (0.79–2.04)	0.318
High	145 (76.7)	127 (72.7)		
Fiber (g/day)	12.1 (2.4–88.3)	12.5 (3.7–87.3)		0.467
Low (*n*, %)	172 (91.0)	160 (90.9)	0.99 (0.48–2.02)	0.974
Normal	17 (9.0)	16 (9.1)		
β-Carotene (μg/day)	0.1 (0–4.8)	0.1 (0–4.2)		0.235^∗^
Low (*n*, %)	153 (81.0)	149 (84.7)	2.82 (0.29–27.39)	0.943
Normal	33 (17.5)	26 (14.8)		0.624
High	3 (1.6)	1 (0.6)		
Thiamine (mg/day)	0.8 (0.2–5.6)	0.8 (0.3–3.7)		0.085^∗^
Low (*n*, %)	166 (87.8)	148 (84.1)	0.73 (0.40–1.33)	0.303
Normal	23 (12.2)	28 (15.9)		
Riboflavin (mg/day)	1.2 (0.3–9.7)	1.3 (0.4–4.6)		0.130^∗^
Low (*n*, %)	117 (61.9)	103 (58.5)	0.87 (0.57–1.32)	0.509
Normal	72 (38.1)	73 (41.5)		
Niacin (mg/day)^¶¶¶^	1.6 (0–40.5)	1.9 (0–30.2)		0.209^∗^
Low (*n*, %)	184 (97.4)	173 (98.3)	1.57 (0.37–6.66)	0.540
Normal	4 (2.1)	3 (1.7)		
High	1 (0.5)	0		
Amino acid (g/day)	105 (72–231)	98.3 (58.5–177)		0.001^∗^
Inadequate (*n*, %)	189 (100)	176 (100)	—	—
Adequate	0	0		
Vitamin K (mg/day)	15.9 (0.2–2025.2)	18.9 (0.1–219.8)		0.336
Low (*n*, %)	168 (88.9)	155 (88.1)	0.92 (0.49–1.76)	0.806
Normal	21 (11.1)	21 (11.9)		
Copper (μg/day)	1300 (300–8200)	1300 (400–5000)		0.614
Low (*n*, %)	189 (100)	176 (100)	0.98 (0.77–1.25)	—
Normal	0	0		
High	0	0		
Sodium (g/day)^††††^	0.58 (0.07–1.56)	0.65 (0.05–4.85)		0.050^∗^
Low (*n*, %)	186 (98.4)	173 (98.3)	0.93 (0.19–4.67)	0.930
Normal	3 (1.6)	1 (0.6)		
High	0	2 (1.1)		

*Note:* Min: minimum; Max: maximum; Fe: ferrum (iron); PUFA: polyunsaturated fatty acid; MUFA: monounsaturated fatty acid; β-carotene: beta-carotene; g: gram; mg: milligram; μg: microgram. The values are presented as the median (min–max) for numerical variables and *n* (%) for categorical variables.

^∗^ *p* value threshold of < 0.25 was used to identify candidate variables for multivariate logistic regression or a *p* value < 0.05 was used to identify statistical significance.

^†^Carbohydrate was dichotomized into low and normal vs. high.

^‡^Total fat was dichotomized into low vs. normal and high.

^§^Fe was dichotomized into low vs. normal and high.

^¶^Zinc was dichotomized into low vs. normal and high.

^††^Magnesium was dichotomized into low vs. normal and high.

^‡‡^Folic acid was dichotomized into low vs. normal and high.

^§§^Vitamin A was dichotomized into low and normal vs. high.

^¶¶^Vitamin D was dichotomized into low vs. normal and high.

^†††^PUFA was dichotomized into 0.6%–1.2% of calorie intake vs. < 0.6% and > 6% of calorie intake.

^‡‡‡^MUFA was dichotomized into low vs. normal and high.

^§§§^Omega-3 was dichotomized into low vs. normal and high.

^¶¶¶^Niacin was dichotomized into low vs. normal and high.

^††††^Sodium was dichotomized into low vs. normal and high.

**Table 4:** Maternal dietary inflammatory index.

Variable	Median (min–max)	*N* (%)
Dietary inflammatory index	−0.133 (−6.15–3.72)	
Q1 (most anti-inflammatory)		94 (25.8)
Q2		85 (23.3)
Q3		84 (23.0)
Q4 (most proinflammatory)		102 (27.9)

*Note:* Min: minimum; Max: maximum. The values are presented as the median (min–max) for numerical variables and *n* (%) for categorical variables.

**Table 5:** Dietary inflammatory index in term and preterm birth.

Variable	Term birth (*n* = 189)	Preterm birth (*n* = 176)	OR (95% CI)	*p* value
Dietary inflammatory index	−0.172 (−6.15–3.72)	−0.131 (−6.00–2.54)		0.522
Q1 (most anti-inflammatory)	49 (25.9)	45 (25.6)	1.09 (0.91–1.31)	0.337
Q2	40 (21.2)	45 (25.6)		
Q3	40 (21.2)	44 (25.0)		
Q4 (most proinflammatory)	60 (31.7)	42 (23.9)		

Abbreviations: CI, confidence interval; OR, odds ratio. The values are presented as the median (min–max) for numerical variables and *n* (%) for categorical variables.

**Table 6:** Multivariate analysis in preterm birth.

Variable	Preterm birth adjusted OR (95% Cl)^a^	*p* value
Maternal characteristics		
Maternal age	0.86 (0.48–1.54)	0.614
Hypertension	0.64 (0.36–1.15)	0.135
Parity	0.75 (0.45–1.25)	0.267
Socioeconomic status	1.21 (0.91–1.61)	0.195
Number of antenatal visits	2.23 (1.34–3.78)	0.001^∗^
Multifetal pregnancy	3.18 (0.91–11.11)	0.069
Micronutrient supplementation	0.64 (0.26–1.57)	0.331
Maternal infections	1.44 (0.83–2.50)	0.191
Vaginal bleeding	3.76 (1.51–9.33)	0.004^∗^
Nutrition intake		
Energy (kcal/day)^†^	1.02 (1.01–1.03)	0.009^∗^
Protein (g/kg/day)^‡^	2.11 (1.15–3.85)	0.015^∗^
Zinc (mg/day)^‡^	0.38 (0.15–0.96)	0.041^∗^
Folic acid (μg/day)^†^	1.00 (0.99–1.01)	0.703
Vitamin A (RDA)^‡^	0.48 (0.26–0.82)	0.006^∗^
Vitamin C (RDA)^‡^	1.19 (0.71–2.00)	0.506
Vitamin B12 (RDA)^†^	1.01 (0.97–1.03)	0.615
PUFA (RDA)^‡^	0.67 (0.41–1.09)	0.110
Omega-3 (mg/day)^‡^	0.94 (0.70–1.25)	0.651
Omega-6 (g/day)^†^	0.92 (0.66–1.30)	0.651
β-Carotene (μg/day)^†^	1.27 (0.90–1.80)	0.170
Thiamine (mg/day)^†^	0.45 (0.13–1.60)	0.219
Riboflavin (mg/day)^†^	1.31 (0.73–2.34)	0.371
Niacin (mg/day)^†^	1.20 (0.77–1.86)	0.419
Amino acid (RDA)^†^	1.02 (0.96–1.05)	0.928
Sodium (g/day)^†^	0.99 (0.99–1.00)	0.087

*Note:* β-Carotene: beta-carotene; PUFA: polyunsaturated fatty acids; kcal: kilocalories; g: gram; mg: milligram; μg: microgram.

Abbreviations: CI, confidence interval; OR, odds ratio.

^∗^ *p* value threshold of < 0.05 was used to identify statistical significance.

^a^Adjusted for maternal age, hypertension, parity, socioeconomic status, number of antenatal visits, multifetal pregnancy, micronutrient supplementation, maternal infection, and vaginal bleeding.

^†^Variables derived from numerical predictors.

^‡^Variables derived from categorical predictors.

## Data Availability

Data described in the manuscript, code book, and analytic code will be made available upon request pending application, approval, and payment.
